# Serum Mitsugumin-53 Level as a Diagnostic Marker in Patients With Acute Coronary Syndrome: A Case-Control Study

**DOI:** 10.7759/cureus.82953

**Published:** 2025-04-24

**Authors:** Sriram M Pattabi, Prashant S Adole, Kolar V Vinod

**Affiliations:** 1 Biochemistry, Jawaharlal Institute of Postgraduate Medical Education and Research, Pondicherry, IND; 2 General Medicine, Jawaharlal Institute of Postgraduate Medical Education and Research, Puducherry, IND

**Keywords:** acute coronary syndrome, creatine kinase-mb, diagnostic marker, heart-type fatty acid binding protein, mitsugumin-53

## Abstract

Background: The high incidence of acute coronary syndrome (ACS) warrants the search for new cardiac biomarkers. Mitsugumin-53 (MG-53) is a myokine involved in cell membrane repair and essential for the function and survival of cardiomyocytes. Therefore, the study assessed the utility of serum MG-53 levels in ACS diagnosis and prognosis.

Method: We enrolled 120 ACS patients as cases and 40 healthy controls as per the inclusion and exclusion criteria in this case-control study. The demographics, present and past history, and physical characteristics were recorded. The chemistry analyzer analyzed routine blood investigations. Serum MG-53 and heart-type fatty acid binding protein (H-FABP as a myocardial injury marker) levels were measured using enzyme-linked immunosorbent assay (ELISA) kits. The Statistical Package for the Social Sciences (SPSS) program (version 26.0, IBM Corp., Armonk, NY) was used for statistical analysis, such as independent t-test, chi-squared test, Pearson’s and Spearman’s correlation, receiver operating characteristic (ROC) curve, and univariate and multivariate logistic regression. A p-value <0.05 was considered statistically significant.

Results: Patients with ACS as cases had higher serum MG-53 and H-FABP levels than controls (p < 0.001). Serum MG-53 level was significantly positively correlated with serum troponin-I (correlation coefficient (r) = 0.520, p < 0.001), creatine kinase-MB (r = 0.298, p = 0.001), and H-FABP (r = 0.792, p < 0.001). The ROC curve showed that the serum MG-53 level had an area under the curve of 0.846 (95% CI: 0.781-0.911, p < 0.001) and sensitivity and specificity of 70%. Multivariate logistic regression depicted that the serum MG-53 level (OR = 1.113, 95% CI: 1.028-1.297, p < 0.001) was significantly associated with ACS risk after adjusting for age, weight, systolic blood pressure, random blood glucose, and serum total cholesterol levels. Multivariate linear regression showed a significant positive association between serum MG-53 and H-FABP levels in patients with different ACS severity and vessel diseases (p < 0.001).

Conclusion: The serum MG-53 level may be considered one of the diagnostic markers in ACS patients. However, further studies need to confirm our findings.

## Introduction

Acute coronary syndrome (ACS) is a condition due to reduced blood circulation to the myocardium secondary to the rupture of atherosclerotic plaque and the formation of a thrombus, resulting first in ischemia and then infarction. Stroke and ischemic heart disease (IHD) are responsible for the majority of deaths due to cardiovascular disease (CVD). It is observed that the age-standardized mortality rate for ACS was higher in low-income regions than in high-income areas globally, and the mortality was higher in men than in women [1]. According to the World Health Organization, India accounts for one-fifth of 17.7 million deaths due to CVD worldwide, especially in the younger population [2]. A higher prevalence of coronary artery disease (CAD) is observed among diabetes mellitus (DM) patients (21.4%) and those living in the rural part of India [3].

Measuring circulating levels of biomarkers is essential for ACS diagnosis, especially in unstable angina (UA) and non-ST-segment elevation myocardial infarction (NSTEMI). Cardiac troponins and creatine kinase-MB (CK-MB) are used widely as markers of myocardial injury. However, there are some drawbacks to these existing markers, like the non-specificity, low sensitivity for cardiac damage, and ambiguity with an electrocardiogram (ECG) [4,5]. Research has been conducted to discover new cardiac biomarkers to help clinicians with ACS diagnosis, prognosis, risk assessment, and treatment [6-8].

Membrane injury and its timely repair are essential for cells’ function and survival. Mitsugumin-53 (MG-53), a tripartite motif protein, is an endogenously secreted myokine that has a role in the repair and regeneration of skeletal and cardiac muscles. MG-53 is involved in excitation-contraction coupling, muscle regeneration, myogenesis, calcium homeostasis, and regulation of mitochondrial functions [9]. Cardiomyocytes are terminally differentiated cells and have limited self-renewal capability. Therefore, membrane repair is important in cardiomyocytes. The mechanism of membrane repair by MG-53 is due to vesicle trafficking and their nucleation at membrane injury, which is thus involved in the resealing process [10].

The significance of MG-53 is observed in various cardiac diseases. For example, MG-53 is important in myocardial protection against ischemia-reperfusion injury [11]. Moreover, MG-53 is essential in ischemic preconditioning (IPC) and postconditioning cardio-protection [12]. The genetic ablation of MG-53 exacerbates myocardial ischemia-reperfusion injury, while overexpression of MG-53 has been protective in animal and cellular models of myocardial injury [13]. Injection of recombinant human MG-53 has reduced ischemia-reperfusion injury, infarct size, and troponin-I release in animal myocardial infarction (MI) models [14]. It was demonstrated that serum MG-53 levels may be valuable biomarkers for CAD diagnosis and may indicate its severity [15].

As MG-53 is involved in the repair of cardiomyocytes, its significance in pre- and post-ischemic conditioning, its protective effects in animal MI, and its correlation with MI severity and limited human studies showed its importance in diagnosing ACS, the present study was conducted to assess the utility of serum MG-53 level in diagnosis and prognosis in ACS patients. The objectives of the study were to collate serum MG-53 levels between ACS patients and healthy controls, to analyze the thrombolysis in myocardial infarction (TIMI) and Global Registry of Acute Coronary Events (GRACE) scores and Killip classes for ACS severity and to determine the association between serum MG-53 level; traditional cardiac markers, i.e., CK-MB, troponin-I, and heart-type fatty acid binding protein (H-FABP); and various risk scores of ACS.

## Materials and methods

Study participants

The case-control pilot study was conducted in the Departments of Biochemistry and Medicine, Jawaharlal Institute of Postgraduate Medical Education and Research (JIPMER), Pondicherry, from January 2022 to December 2023. The Institutional Ethics Committee on human participants reviewed and approved the study (JIP/IEC/2022/009, dated 09/02/2022), and the procedures were according to the Helsinki Declaration guidelines. All study participants gave written informed consent. The study included 120 ACS patients, including UA, NSTEMI, and ST-segment elevation myocardial infarction (STEMI) as cases. STEMI is diagnosed in patients with acute onset of chest discomfort and ECG findings of new ST-segment elevation at the J-point in two contiguous leads, with the cut-points ≥ 0.1 mV in all leads other than leads V2 to V3 and for leads V2 to V3, ≥ 2 mm in men ≥ 40 years; ≥ 2.5 mm in men < 40 years, or ≥1.5 mm in women regardless of age. NSTEMI is diagnosed in patients with ACS symptoms and elevation of troponins but without ECG changes similar to STEMI. Elevation of troponin I levels with a dynamic early change is used to diagnose NSTEMI patients and differentiate between NSTEMI and UA [16]. Forty apparently healthy controls were selected from friends, neighbors, or relatives of the cases with similar age (±3) and gender, having normal ECG and serum CK-MB levels from JIPMER, Puducherry, to ensure the proper representation of the general population. Patients with muscle, bone, heart, kidney, and peripheral vessel diseases were excluded from the study based on history, clinical examination, and routine biochemical investigations. 

Data collection and laboratory investigations

General characteristics, including age, gender, smoking, alcoholism, and personal and family history of medical illness like DM, hypertension (HTN), and IHD, were recorded. The weight, height, body mass index (BMI), waist circumference, and seated systolic and diastolic blood pressure were measured. The TIMI and GRACE risk scores determined the major adverse cardiac events (MACE) in ACS patients at the time of hospital admission by the treating doctor [17]. The occurrence of MACE, including non-fatal stroke, heart failure, reinfarction, and mortality, was determined in the study participants. The TIMI score assesses seven elements, while the GRACE score assesses eight [18]. The Killip classification consists of four classes and stratifies ACS patients as per the severity of their post-MI heart failure [19]. The study utilized different ACS risk scores so that the severity of ACS patients was accurately determined, and the outcome (non-fatal stroke, heart failure, reinfarction, and mortality) in ACS patients could be determined. Peripheral venous blood (5 mL) was collected from ACS patients at admission and from healthy controls at the time of inclusion in the study under strict aseptic precautions. The serum was obtained after centrifugation and used to measure random blood sugar, cardiac markers, kidney function tests, liver function tests, and lipid profiles on a fully automated chemistry analyzer (AU 5800, Beckman) by appropriate methods. Hematological investigations, like hemoglobin, platelets, and white blood cell count, were measured using a cell counter (XN-350TM, Sysmex America, Inc., IL 60069). Serum MG-53 (GENLISATM, Cerritos, CA 90703, USA) and H-FABP (Wuhan, China) levels were assessed by enzyme-linked immunosorbent assay (ELISA) kits. The quality control department of the manufacturer validated all ELISA kits. The known concentrations (standards) of each analyte were analyzed, and the standard curve was prepared, which showed good linearity with an R2 of more than 0.9954. The standards were run along with samples, which showed reproducibility of results. Each ELISA kit has lower sensitivity, a more comprehensive measuring range, good recovery and stability, and < 10% intraday and interday variability. 

Statistical analysis

The Statistical Package for the Social Sciences (SPSS) program (version 26.0, IBM Corp., Armonk, NY) was used for statistical analysis. The Kolmogorov-Smirnov test assessed the normality of data. Normally distributed continuous variables were expressed as mean ± standard deviation (SD) and compared using the independent sample Student’s t-test. Non-normally distributed variables were expressed as median (interquartile range) and compared using the Mann-Whitney test. Categorical variables were expressed as numbers (percentages) and compared using the Chi-square test of homogeneity or Fisher’s exact test. The comparison of serum MG-53 levels among more than two groups was analyzed using a one-way analysis of variance or the Kruskal-Wallis test. Pearson and Spearman’s correlation coefficients were used to find the correlation between study parameters (MG-53 and H-FABP) and clinical, biochemical, and patient characteristics. Multiple linear regression was analyzed to evaluate the relationship between serum MG-53 and H-FABP levels (an indicator of myocardial injury) in various groups after adjusting for age, weight, systolic blood pressure, random blood sugar, and serum total cholesterol level, as they showed significant correlations with serum MG-53 levels. The serum MG-53 diagnostic predictive values (OR and 95% CI) for ACS were assessed by univariate and multivariate binary logistic regressions. The linear and logistic regressions were based on linearity testing. The diagnostic test characteristics, like sensitivity, specificity, cut-off value, and the area under the curve, were determined using the receiver operating characteristic (ROC) curve analysis to distinguish between ACS patients and healthy controls. All statistical tests were performed at a 95% significance level.

## Results

General characteristics and laboratory investigations 

**Figure 1:**
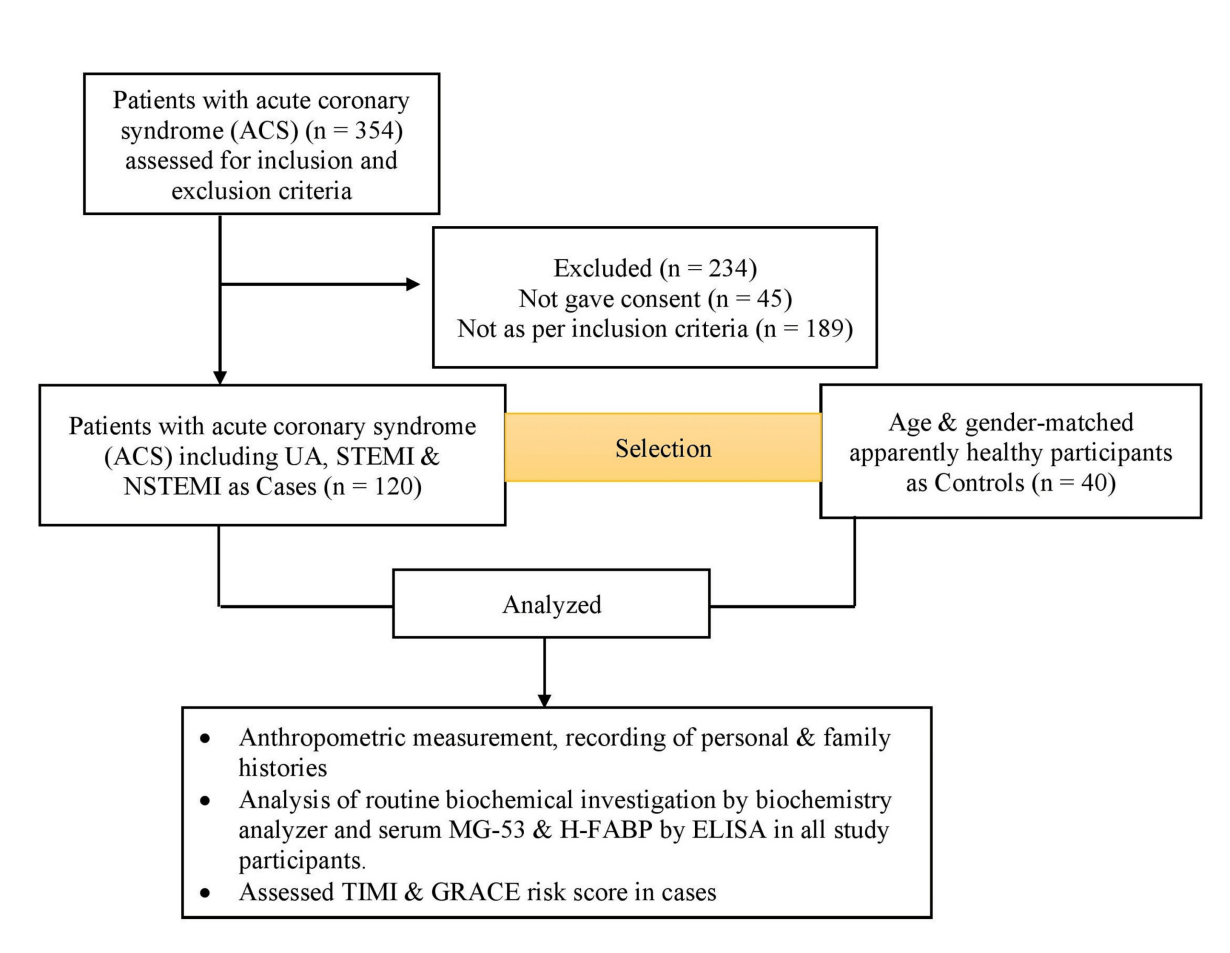
A flowchart of the selection of the study participants.

A total of 354 patients with ACS were screened. Out of 354, only 120 ACS as cases were included in the study as per the inclusion and exclusion criteria. The study included 40 age and gender-matched, apparently healthy participants as controls. A flowchart of the selection of study participants is shown in Figure 1. 

The essential features of the study participants are shown in Table 1. 

**Table 1:** Clinical and biochemical properties of the study population ACS, acute coronary syndrome; DM, diabetes mellitus; HTN, hypertension; IHD, ischemic heart disease; STEMI, ST-segment elevation myocardial infarction; NSTEMI, non-ST-segment elevation myocardial infarction; BMI, body mass index; HDL-C, high-density lipoprotein cholesterol; LDL-C, low-density lipoprotein cholesterol; BUN, blood urea nitrogen; CK-total, creatine kinase-total; CK-MB, creatine kinase-MB. Data are expressed as median (interquartile range), mean ± standard deviation or number (percentage) as appropriate. Significance at 0.05 level when compared between the two groups using t-test for normally distributed continuous variables, the Mann-Whitney U test for non-normally distributed variables, and the Chi-square test of homogeneity or Fisher’s exact test for categorical variables.

Parameters	Patients with ACS, Cases (n = 120)	Healthy controls (n = 40)	p-value
Age (y)	55±11	54±6	0.624
Male/ Female (n/n)	99/21	33/7	0.806
Smoker (n,%)	49 (40.8)	1 (2.5)	< 0.001
Alcoholics (n,%)	56 (46.6)	8 (20)	0.005
Presence of DM (n,%)	54 (45)	8 (20)	0.008
Presence of hypertension (n,%)	31 (25.8)	7 (17.5)	0.391
Presence of ischemic heart disease (n,%)	18 (15)	4 (10)	0.597
Family history of DM (n,%)	38 (31.6)	8 (20)	0.226
Family history of HTN (n,%)	35 (29.6)	7 (17.5)	0.212
Family history of IHD (n,%)	21 (17.5)	4 (10)	0.322
Time between onset of chest pain and admission (h)	24.82±6.37	-	-
Type of ACS			
STEM (n,%)	104 (86.67)	-	-
NSTEMI (n,%)	10 (8.33)	-	-
Unstable angina (n,%)	06 (5)	-	-
Type of vessel diseases (Out of 73)			
Single-vessel disease (n,%)	36 (49.31)	-	-
Double-vessel disease (n,%)	24 (32.87)	-	-
Triple-vessel disease (n,%)	13 (17.82)	-	-
Weight (kg)	73.80±11.94	65.23±12.23	< 0.001
Height (cm)	1.59±0.09	1.67±0.09	0.359
Waist circumference (inch)	35.50±5.40	34.31±4.30	0.104
BMI (kg/m^2^)	25.56±4.45	26.38±3.15	0.285
Systolic blood pressure (mm Hg)	128 (110-140)	125 (116-129)	0.288
Diastolic blood pressure (mm Hg)	80 (70-90)	79 (72-85)	0.600
Pulse rate (beats/min)	87.36±19.92	76.90±4.90	0.001
Hemoglobin (g/dL)	13.62±2.77	14.01±1.36	0.396
White blood cell count (´10^3^/mL)	11.18 (9.26-13.27)	7.05 (6.07-8.45)	< 0.001
Platelets (´ 10^3^/mL)	260.12±79.16	280.32±64.30	0.146
Random blood glucose (mg/dL)	157 (111-276)	67.5 (60-79)	< 0.001
Total cholesterol (mg/dL)	183.12±48.76	205.00±45.28	0.013
Triglycerides (mg/dL)	127 (85-174)	107 (75- 60)	0.220
LDL-C (mg/dL)	113.90±30.70	132.80±36.42	0.002
HDL-C (mg/dL)	39±8.19	50.2±13.07	< 0.001
BUN (mg/dL)	28 (21-37)	21 (17-26)	0.010
Creatinine (mg/dL)	0.90 (0.75-1.05)	0.90 (0.77-0.97)	0.435
CK-total (IU/L)	490 (324-734)	176 (116-227)	< 0.001
CK-MB (IU/L)	60 (39-78)	19 (14-22)	< 0.001
Troponin-I (ng/L)	534 (342-677)	7 (5-8)	< 0.001

Cases and controls were matched for age and gender. Cases had significantly higher smokers, alcoholics, and the percentage of patients with DM compared to controls (p < 0.05). No significant difference was found with respect to the percentage of patients with HTN and IHD and family history of DM, HTN, and IHD between cases and controls. The time between the onset of chest pain and admission among cases was 24.82 ± 6.37 hours. Out of 120 ACS patients, 104 (86.67%) had STEMI, 10 (8.33%) had NSTEMI, and six (5%) had UA. Coronary angiography was available only for 73 ACS patients due to logistical issues. Out of 73 ACS patients, 36 (49.31%) had single-vessel disease (SVD), 24 (32.87%) had double-vessel disease (DVD), and 13 (17.82%) had triple-vessel disease (TVD). 

Cases had significantly lower serum total cholesterol, LDL-C, and HDL-C levels than controls (p < 0.05). Moreover, cases had higher weight, pulse rate, white blood cell count, random blood sugar, and BUN than controls. However, no significant difference was found between the two groups regarding height, waist circumference, BMI, systolic blood pressure, diastolic blood pressure, hemoglobin, platelet count, serum triglyceride, and creatinine levels. Out of 120 ACS patients, 59 (49.17%) were low-risk, 48 (40%) were intermediate-risk, and 13 (10.83%) had high-risk TIMI scores. Out of 120 ACS patients, 51 (42.5%) were low-risk, 47 (39.17%) were intermediate-risk, and 22 (10.33%) had high-risk GRACE scores. Out of 120 ACS patients, 92 (76.7%) had Killip class 1, 9 (7.5%) had Killip class 2, 10 (8.3%) had Killip class 3, and 9 (7.5%) had Killip class 4. 

Correlation and linear regression

**Figure 2:**
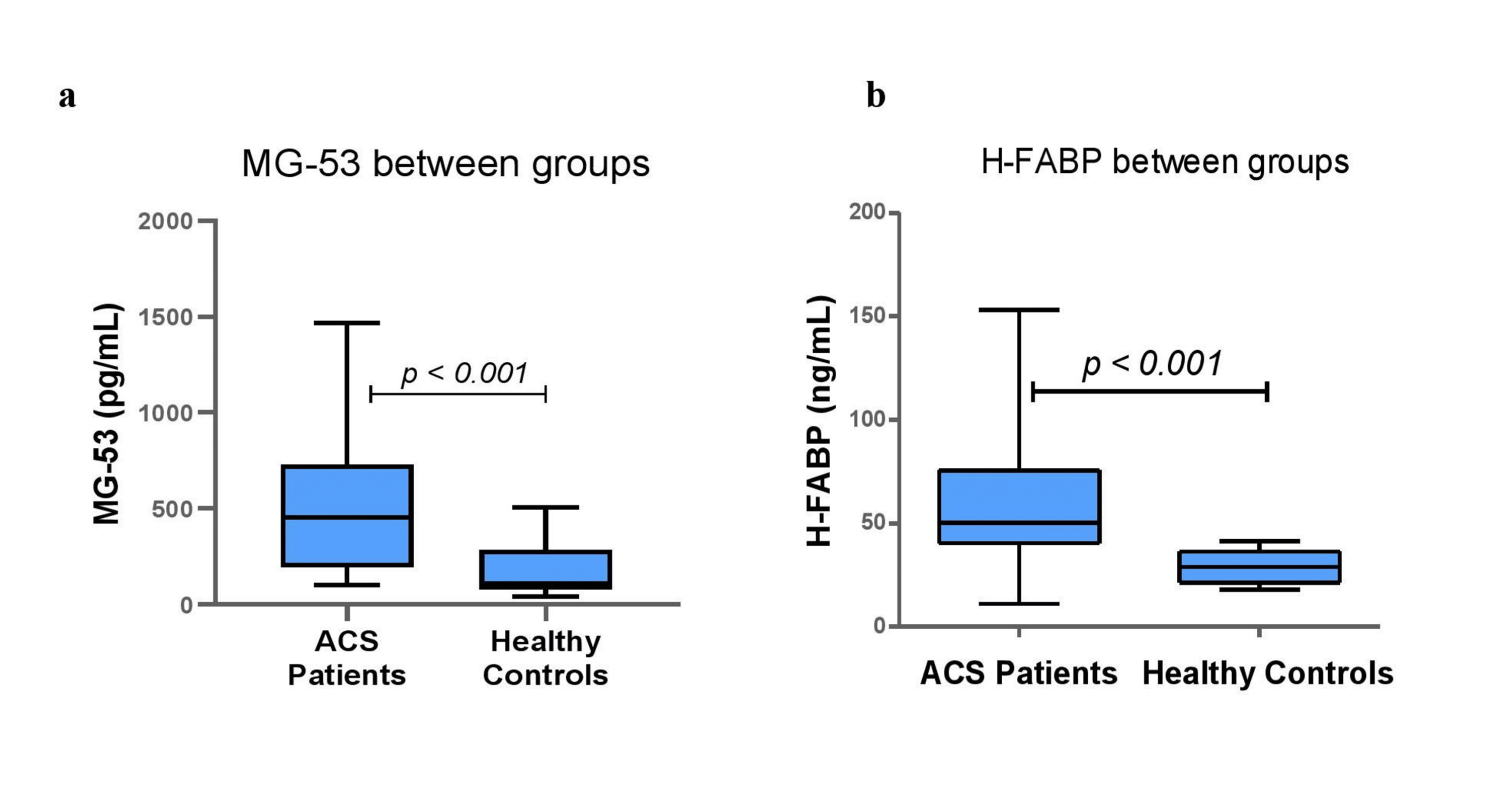
Comparison of serum (a) mitsugumin-53 (MG-53) and (b) heart-type fatty acid binding protein (H-FABP) levels between patients with acute coronary syndrome (ACS) and healthy controls.

A significantly higher serum MG-53 level was observed in cases than in controls (451.95 (197.47 to 729.97) vs. 109.66 (80.88 to 282.08) pg/mL, p < 0.001; Figure 2a). Moreover, a significantly higher serum H-FABP level was observed in cases than in controls (50.33 (40.33 to 75.55) vs. 28.85 (21.34 to 36.38) ng/mL, p < 0.001; Figure 2b).

**Figure 3:**
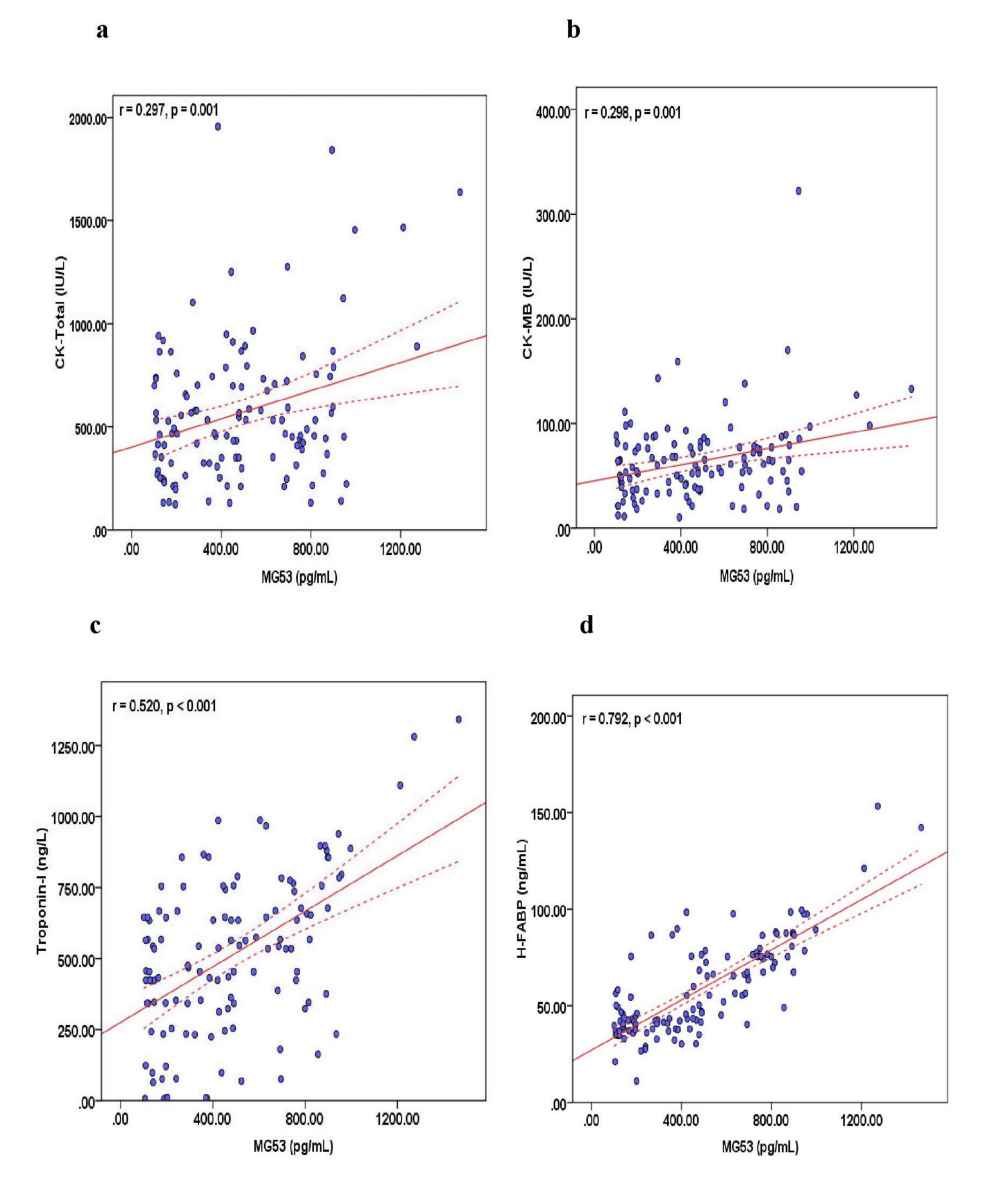
Correlation of serum MG-53 levels with serum (a) CK-total, (b) CK-MB, (c) troponin-I, and (d) H-FABP levels in acute coronary syndrome (ACS) patients. Each symbol represents as individual patient.; red lines are 95% confidence interval.

No significant difference was found in the serum MG-53 level between patients with low-risk and intermediate-risk TIMI scores (p = 0.463) and between low-risk and high-risk TIMI scores (p = 0.935). Moreover, no significant difference was found in the serum MG-53 level between patients with low-risk and intermediate-risk GRACE scores (p = 0.950) and between low-risk and high-risk GRACE scores (p = 0.958). No significant difference was found in the serum MG-53 level between patients with SVD and DVD ACS (p = 0.433) and between SVD and TVD ACS (p = 0.182). Moreover, no significant difference was found in the serum MG-53 level between patients with Killip 1 class and 2 class (p = 0.323), 1 class and 3 class (p = 0.175), and 1 class and 4 class (p = 0.991). In ACS patients, the serum MG-53 level was significantly positively correlated with age (r = 0.526, p = 0.010), the time between the onset of chest pain and admission (r = 0.322, p = 0.029), weight (r = 0.482, p = 0.034), systolic blood pressure (r = 0.357, p = 0.032), random blood sugar (r = 0.432, p = 0.012), serum total cholesterol (r = 0.435, p = 0.001), CK-total (r = 0.297, p = 0.001; Figure 3a), CK-MB (r = 0.298, p = 0.001; Figure 3b), troponin-I (r = 0.520, p < 0.001; Figure3c), and H-FABP (r = 0.792, p < 0.001; Figure 3d) and negatively associated with waist circumference (r = -0.278, p = 0.002), platelets (r = -0.208, p = 0.023), as shown in Table 2. Similarly, significant correlations were also observed between serum MG-53 levels and cardiac markers of MI in STEMI and NSATMI patients.

**Table 2:** Correlation of serum MG-53 and H-FABP levels with other variables among ACS patients. MG-53, mitsugumin-53; H-FABP, heart-type fatty acid binding protein; ACS, acute coronary syndrome; DM, diabetes mellitus;  HTN, hypertension; IHD, ischemic heart disease; BMI, body mass index; HDL-C, high-density lipoprotein cholesterol; LDL-C, low-density lipoprotein cholesterol; BUN, blood urea nitrogen; CK-total, creatine kinase-total; CK-MB, creatine kinase-MB; TIMI, thrombolysis in myocardial infarction; GRACE, global registry of acute coronary events. Correlation assessed using Pearson and  Spearman’s correlation coefficient.

Parameters n = 120	MG-53		H-FABP	
	r	p-value	r	p-value
Age	0.526	0.010	0.4351	0.023
Gender	0.031	0.738	0.033	0.720
Presence of DM	0.116	0.207	0.074	0.425
Presence of HTN	-0.016	0.859	-0.045	0.628
Presence of IHD	-0.092	0.320	-0.051	0.577
Onset of chest pain	0.322	0.029	0.383	0.032
Weight	0.482	0.034	0.396	0.029
Height	-0.056	0.547	0.003	0.971
Waist circumference	-0.278	0.002	-0.212	0.020
BMI	-0.053	0.565	-0.052	0.572
Systolic blood pressure	0.357	0.032	0.432	0.021
Diastolic blood pressure	0.095	0.304	0.131	0.153
Pulse rate	-0.161	0.080	-0.072	0.432
Hemoglobin	-0.028	0.403	-0.094	0.798
Platelets	-0.208	0.023	-0.107	0.244
Random blood glucose	0.432	0.012	0.342	0.023
Total cholesterol	0.435	0.001	0.421	0.001
Triglycerides	0.044	0.632	0.130	0.156
HDL	-0.094	0.308	-0.047	0.610
LDL	0.123	0.165	0.093	0.311
BUN	-0.033	0.721	-0.012	0.895
Creatinine	-0.062	0.504	-0.038	0.679
CK-total	0.297	0.001	0.337	< 0.001
CK-MB	0.298	0.001	0.355	< 0.001
Troponin-I	0.520	< 0.001	0.738	< 0.001
TIMI score	-0.023	0.804	-0.068	0.460
GRACE score	0.015	0.867	-0.063	0.496
Killip classes	0.035	0.704	-0.056	0.545

By considering serum H-FABP level as a marker of acute myocardial injury, the study analyzed the relationship between serum MG-53 and H-FABP levels using the multivariate linear regression analysis adjusted for age, weight, systolic blood pressure, random blood sugar, and serum total cholesterol level. It showed that the serum MG-53 level was significantly correlated with serum H-FABP level in patients with low-, intermediate-, and high-risk TIMI and GRACE scores and SVD, DVD, and TVD, and KILLIP 1, 2, 3, and 4 risk scores, as shown in Table 3.

**Table 3:** Linear regression analysis of serum H-FABP level in various groups H-FABP, heart-type fatty acid binding protein; TIMI, thrombolysis in myocardial infarction; MG-53, mitsugumin-53; GRACE, global registry of acute coronary events; SVD, single-vessel disease; DVD, double-vessel disease; TVD, triple-vessel disease.

Variables	Patients with low-risk TIMI score			Patients with intermediate-risk TIMI score			Patients with high-risk TIMI score		
	b	t	p-value	b	t	p-value	b	t	p-value
Age	-0.076	-0.868	0.389	0.247	2.845	0.007	0.299	3.381	0.007
Weight	-0.036	-0.393	0.696	-0.049	-0.523	0.604	-0.037	-0.285	0.782
Systolic blood pressure	0.043	0.494	0.624	0.057	0.597	0.554	- 0.050	- 0.387	0.707
Random blood sugar	0.025	0.287	0.775	0.048	0.518	0.607	-0.054	-0.422	0.682
Total cholesterol	-0.019	-0.213	0.832	0.009	0.092	0.927	0.193	1.463	0.174
MG-53	0.753	8.634	< 0.001	0.781	8.473	< 0.001	0.912	7.391	< 0.001
Variables	Patients with low-risk GRACE score			Patients with intermediate-risk GRACE score			Patients with high-risk GRACE score		
	b	t	p-value	b	t	p-value	b	t	p-value
Age	-0.137	-1.494	0.142	0.211	2.195	0.033	0.009	0.087	0.931
Weight	-0.042	-0.440	0.662	-0.097	-0.965	0.340	0.054	0.526	0.605
Systolic blood pressure	-0.007	-0.069	0.945	0.146	1.465	0.150	-0.069	-0.666	0.514
Random blood sugar	0.103	1.113	0.271	-0.014	-0.138	0.891	-0.058	-0.558	0.584
Total cholesterol	0.112	1.204	0.235	-0.139	-1.404	0.167	0.125	1.180	0.253
MG-53	0.759	8.149	< 0.001	0.740	7.377	< 0.001	0.892	8.805	< 0.001
Variables	Patients with SVD			Patients with DVD			Patients with TVD		
	b	t	p-value	b	t	p-value	b	t	p-value
Age	-0.156	-1.206	0.236	-0.060	-0.438	0.666	-0.129	-0.513	0.619
Weight	-0.031	-0.232	0.818	0.103	0.762	0.454	-0.397	-1.807	0.101
Systolic blood pressure	0.110	0.836	0.409	-0.019	-0.132	0.896	-0.333	-1.045	0.320
Random blood sugar	0.023	0.178	0.860	0.070	0.520	0.609	0.082	0.257	0.803
Total cholesterol	0.046	0.341	0.735	-0.065	-0.482	0.635	0.502	2.511	0.023
MG-53	0.658	5.098	< 0.001	0.783	5.903	< 0.001	0.600	2.486	< 0.001

Logistic regression analysis of factors correlated with ACS

On univariate logistic regression, age (OR = 0.908, 95% CI = 0.872-0.946, p < 0.001), weight (OR = 1.058, 95% CI = 1.025-1.091, p < 0.001), systolic blood pressure (OR = 0.985, 95% CI = 0.971-1.000, p = 0.043), random blood sugar(OR = 0.932, 95% CI = 0.906-0.958, p < 0.001), total cholesterol (OR = 1.009, 95% CI = 1.002-1.017, p = 0.017), and serum MG-53 (OR = 0.993, 95% CI = 0.990-0.996, p < 0.001) levels were associated ACS in the study participants. However, after using these parameters in multivariate logistic regression, we found that weight (OR = 1.065, 95% CI = 1.002-1.131, p < 0.001), random blood sugar (OR = 1.073, 95% CI = 1.012-1.232, p < 0.001), and serum MG-53 levels (OR = 1.113, 95% CI = 1.028-1.297, p < 0.001) were independently associated with ACS in the study participants, as shown in Table 4.

**Table 4:** Logistic regression analysis of factors to ACS outcome in the study participants. ACS, acute coronary syndrome; OR, odds ratio; CI, confidence interval; MG-53, mitsugumin-53.

Variables	Univariate			Multivariate		
	OR	95% CI	p-value	Adjusted OR	95% CI	p-value
Age	0.908	(0.872-0.946)	<0.001	-	-	0.423
Weight	1.058	(1.025-1.091)	<0.001	1.065	(1.002-1.131)	<0.001
Systolic blood pressure	0.985	(0.971-1.000)	0.043	-	-	0.321
Random blood sugar	0.932	(0.906-0.958)	<0.001	1.073	(1.012-1.232)	<0.001
Total cholesterol	1.009	(1.002-1.017)	0.017	-	-	0.532
MG-53	0.993	(0.990-0.996)	<0.001	1.113	(1.028-1.297)	<0.001

ROC curve analysis of serum MG-53 and H-FABP levels to predict ACS

**Figure 4:**
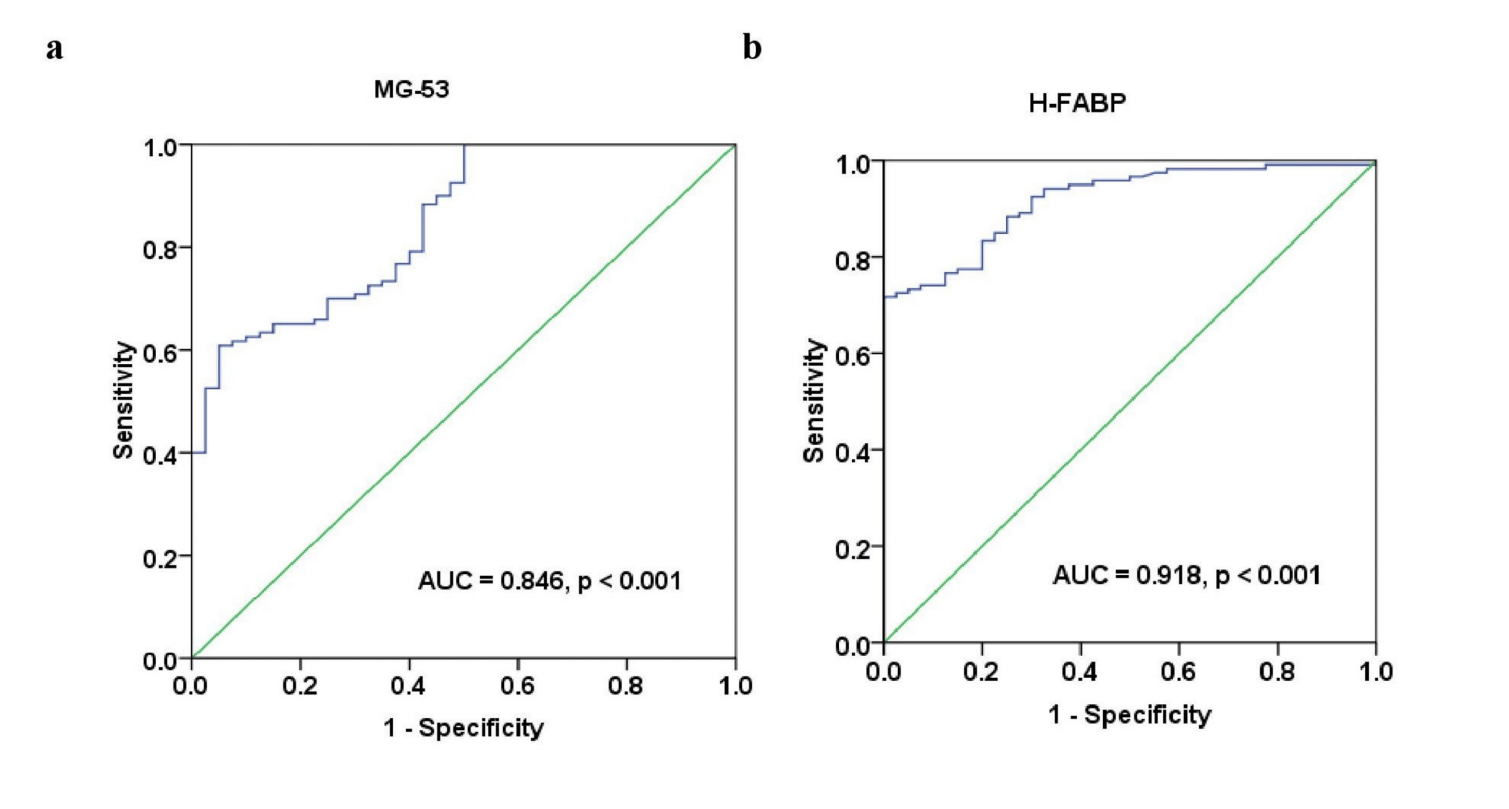
Receiver operating characteristic curve analysis of serum (a) MG-53 and (b) H-FABP levels to predict acute coronary syndrome (ACS) in the study participants.

ROC curve analysis revealed that the optimum cut-off value of serum MG-53 level for predicting ACS was 247.10 pg/mL with sensitivity and specificity of 70% (CI = 67-73) and an AUC of 0.846 (95% CI = 0.781-0.911, p < 0.001), as shown in Figure 4a. Also, the significant cut-off value of the serum H-FABP level for predicting ACS was 37.99 pg/mL with sensitivity and specificity of 80% (CI = 78-82) and with an AUC of 0.918 (95% CI = 0.876-0.960, p < 0.001), as shown in Figure 4b. Delong’s test demonstrated that serum H-FABP levels have significantly different AUC than serum MG-53 levels (p < 0.05).

## Discussion

Considering the increasing prevalence and mortality of ACS patients and the limitations of the traditional cardiac markers for diagnosing ACS, the study evaluated the utility of the serum MG-53 level in the diagnosis and prognosis of ACS patients. The study found that the serum MG-53 level may have potential diagnostic utility for ACS diagnosis.

Myokines are the polypeptides released from muscle fibers during contractions or exercise. They influence the metabolic pathways in the liver, pancreas, and adipose tissue, thermogenic activity, and the development of CVD. Hence, myokines are used in the diagnosis and risk stratification in patients with MI, heart failure, atherosclerosis, DM, and HTN [20]. The present study included 120 ACS patients as cases and 40 healthy controls to elucidate the significance of the serum MG-53 level, one of the myokines, for ACS diagnosis and prognosis. The present study showed a higher percentage of smokers, alcoholics, and DM in cases than controls, which may have contributed to ACS precipitation; however, other risk factors like age, gender, obesity, history of HTN, and family history of DM, HTN, and IHD were similar among them. The predominance of STEMI patients over NSTEMI and UA among cases indicates higher atherosclerosis and its complications. Also, it is due to the early referral of STEMI patients to the tertiary center for urgent revascularization.

The study showed a higher level of serum MG-53 in ACS patients than in healthy controls. This finding is in accordance with findings from various animal and human studies in the literature [14,15,21]. For example, a higher serum MG-53 level was shown in 222 patients with stable coronary disease and was highest in 212 patients with AMI compared to 205 patients without coronary artery stenosis [15]. A higher serum MG-53 level signifies cardioprotective effects due to its role in ischemic preconditioning and postconditioning, repair of membrane injury, and reduced atrial fibrosis and inflammation. The repair of cardiomyocytes due to ischemic injury is the primary mechanism of cardioprotection by MG-53 due to the limited self-renewal capacity of cardiomyocytes [10,13]. The beneficial and cardioprotective role of MG-53 has been supported by animal studies after MG-53 supplementation. For example, the cardioprotective effects of recombinant MG-53 were demonstrated through inhibition of NF-kB-mediated inflammation, reduction of apoptotic cell death, and oxidative stress in the heart of aged mice, thus preventing age-related decline in cardiac function [22]. The recombinant MG-53 improved cardiac functions in mouse and pig models of MI by reducing mitochondrial reactive oxygen species production, preserving mitochondrial membrane potential, and decreasing mitophagy in cardiomyocytes [23]. Most of the MG-53’s functions were from animal studies, and there were limited human studies to support the findings, so the transition of MG-53’s functions to humans should be taken cautiously.

The present study showed a significant association between serum MG-53 levels and markers of MI, like serum CK-MB, cardiac troponin-I, and H-FABP levels, among ACS patients. The association between serum MG-53 and H-FABP levels was confirmed by linear regression in patients with different TIMI and GRACE risk scores, Killip classes, and vessel disorders after adjusting for age, weight, systolic blood pressure, random blood sugar, and serum total cholesterol level. Cardiac troponins, i.e., T or I, are proteins associated with muscle contractions. Cardiac troponins are the preferred markers for diagnosing AMI [24]. The significance of cardiac biomarkers is greater in NSTEMI patients than in STEMI patients for early diagnosis. Also, H-FABP, a cytoplasmic protein, is mainly present in the myocardium and is involved in fatty acid transport and metabolism. Due to myocardium specificity, its level of circulation increases after myocardial injury. Therefore, it is considered a biomarker of myocardial injury and is helpful for early detection and prognosis of AMI [25,26]. The significant association between serum MG-53 and troponin-I or H-FABP levels showed that the serum MG-53 level may be considered a diagnostic marker for ACS. Combining serum MG-53 levels with traditional cardiac biomarkers for ACS diagnosis might have better sensitivity and specificity than individual biomarkers. The positive association of the serum MG-53 level with the time between the onset of chest pain and admission signifies increased MG-53 expression in line with higher myocardial ischemia. As information about MG-53 release kinetics is unavailable in humans, we could not conclude the extent of higher MG-53 expression among ACS patients and correlate it with other biomarkers' release kinetics. Also, univariate and logistic regressions concluded that weight, random blood sugar, and serum MG-53 level were associated with ACS. ROC analyses revealed that the serum MG-53 level had good AUC, sensitivity, and specificity for ACS diagnosis, comparable to the serum H-FABP level. The ROC analysis was done only on study samples after internal validation, such as bootstrapping, which limits its generalizability to other populations. 

Also, the current study showed higher random blood sugar levels in ACS patients than in healthy controls and a positive association between serum MG-53 levels and random blood sugar levels in ACS patients. However, in the literature, multiple studies have shown the contradictory effects of MG-53 on insulin sensitivity. The inhibition of insulin signaling was demonstrated in mice by MG-53, which led to insulin resistance and metabolic syndrome [27]. However, no association between MG-53 and glycemic control was seen in a case-control study with 107 type 2 diabetes (T2D) patients and 105 participants without insulin resistance-related disease [28]. Therefore, DM contributions to ACS precipitation may not be accurate in the current study participants.

Although we assumed that serum MG-53 levels would increase in ACS patients with high TIMI or GRACE scores compared to ACS patients with low scores, the study found no association between the serum MG-53 level, TIMI, GRACE scores, and Killip classes. Also, no significant difference in the serum MG-53 level was found in patients with various vessel diseases, TIMI and GRACE scores, and Killip classes. However, many studies showed that the serum MG-53 level was considered a prognostic factor in AMI patients. For example, the serum MG-53 level was used as a prognostic indicator of MACEs in patients with AMI, independent of conventional risk factors [29]. However, few studies lack the association between MG-53 and MI prognosis. For example, the serum MG-53 level was not associated with cardiovascular risk and did not predict long-term mortality among 296 Caucasian T2D patients [30]. The non-significant association between the serum MG-53 level and various ACS risk scores in the current study may be due to selection bias (higher STEMI patients) or limited sample size.

This study has a few limitations. First, it was cross-sectional, so it is challenging to assess the cause-and-effect link between serum MG-53 and ACS. Furthermore, we lacked information about the later occurrence of ACS in healthy participants. The NSTEMI patients were under-represented in the study, where the utility of cardiac markers is high. No association between ACS risk scores and serum MG-53 levels should be verified through further prospective studies. In addition, a larger cohort may be required to validate the results due to the relatively limited sample size and the uneven number of cases in each category of ACS severity. As most of MG-53’s functions were based on the animal studies and there are limited human studies to support its functions, the transition to human beings may require further new prospective studies on a large sample size. Multilinearity or confounders were not considered during statistical analysis. Cross-validation techniques were not employed in this study, and the lack of such validation may result in overfitting.

## Conclusions

As the prevalence of ACS is increasing in the population and there are limitations with available cardiac markers for ACS diagnosis, a novel biomarker becomes essential for early diagnosis and prognosis of ACS patients. We found that ACS patients had higher serum MG-53 levels than healthy controls. Also, the serum MG-53 level was associated with circulating cardiac markers of MI, like CK-MB, troponin-I, and H-FABP. Linear and logistic regression confirmed the association between serum MG-53 and ACS after adjusting for confounding factors. The ROC curve analysis revealed that serum MG-53 levels distinguished ACS patients with reasonable specificity, sensitivity, and the AUC comparable with serum H-FABP levels. Therefore, serum MG-53 levels may have potential utility in the diagnosis of ACS patients. The small sample size of our study may limit both the statistical power and the generalizability of the findings. Cohort studies or multi-centre studies with a larger sample size are warranted to determine their predictive value in the future and improve their generalizability.
